# The Maternal and Child Health (MCH) Handbook in Mongolia: A Cluster-Randomized, Controlled Trial

**DOI:** 10.1371/journal.pone.0119772

**Published:** 2015-04-08

**Authors:** Rintaro Mori, Naohiro Yonemoto, Hisashi Noma, Tumendemberel Ochirbat, Emma Barber, Gochoo Soyolgerel, Yasuhide Nakamura, Oyun Lkhagvasuren

**Affiliations:** 1 Department of Health Policy, National Center for Child Health and Development, Tokyo, Japan; 2 Department of Psychopharmacology, National Center of Neurology and Psychiatry, Tokyo, Japan; 3 Department of Data Science, The Institute of Statistical Mathematics, Tokyo, Japan; 4 Department of Global Health Policy, Graduate School of Medicine, University of Tokyo, 7–3–1 Hongo, Bunkyo-ku, Tokyo, Japan; 5 Medical Care Policy Implementation and Coordination, Ministry of Health, Ulaanbaatar, Mongolia; 6 Department of International Collaboration, Graduate School of Human Sciences, Osaka University, Osaka, Japan; 7 Faculty of Health Science, Simon Fraser University, Vancouver, Canada; Fondazione IRCCS Ca' Granda Ospedale Maggiore Policlinico, Università degli Studi di Milano, ITALY

## Abstract

**Objective:**

To assess the effectiveness of the Maternal and Child Health (MCH) handbook in Mongolia to increase antenatal clinic attendance, and to enhance health-seeking behaviors and other health outcomes.

**Methods:**

A cluster randomized trial was conducted using the translated MCH handbook in Bulgan, Mongolia to assess its effectiveness in promoting antenatal care attendance. Pregnant women were recruited from 18 randomly allocated districts using shuffled, sealed envelopes. The handbook was implemented immediately for women at their first antenatal visit in the intervention group, and nine months later in the control group. The primary outcome was the number of antenatal care visits of all women residing in the selected districts. Cluster effects were adjusted for using generalized estimation equation. Masking was not possible among care providers, pregnant women and assessors.

**Findings:**

Nine districts were allocated to the intervention group and the remainder to the control group. The intervention group (253 women) attended antenatal clinics on average 6•9 times, while the control group (248 women) attended 6•2 times. Socioeconomic status affected the frequency of clinic attendance: women of higher socioeconomic status visited antenatal clinics more often. Pregnancy complications were more likely to be detected among women using the handbook.

**Conclusion:**

The MCH handbook promotes continuous care and showed an increase in antenatal visits among the intervention group. The intervention will help to identify maternal morbidities during pregnancy and promote health-seeking behaviors.

**Trial Registration:**

UMIN Clinical Trial Registry UMIN000001748

## Introduction

Maternal and child health continues to present a significant public health challenge in Mongolia. Despite a marked improvement in the maternal and neonatal mortality ratios over the past 20 years, with 89·6 per 100,000 births in 2007[1] and 14 per 1,000 births during 2001–2003[2], respectively, as well as a decline in the mortality of older children,[1] the quality of antenatal care is still low[3] and complications during pregnancy remain a significant hurdle for improving maternal health in Mongolia.[4, 5] Effective interventions to enhance maternal and child health outcomes are crucial to address these challenges and to maintain the achievement of health-related Millennium Developmental Goals (MDGs) 4 and 5.[6]

An ongoing challenge for researchers and health professionals is how to deliver effective interventions to reduce maternal and neonatal mortality in resource-limited settings. Effective maternal health interventions should aim to encourage health-seeking behaviors among pregnant women and increase their maternal knowledge.[7] As the role of health workers is to promote healthcare-seeking behaviors and initiate preventive action,[8] a health record book such as Japan’s Maternal and Child Health (MCH) handbook[9] could be used as an effective tool by community healthcare workers and professional hospital staff to enhance client–provider communication about health,[10, 11] raise health awareness, and identify complications earlier in the pregnancy.[11, 12]

The purpose of introducing the handbook to Mongolia, which was proposed by the Mongolian Ministry of Health, was to increase antenatal visits and enhance client-provider communication during pregnancy to improve long-term health outcomes for mother and child. The handbook was first considered by the Mongolian government as a key intervention in maternal and child health in 2007,[13] and our study initiated the national adoption of the MCH handbook in Mongolia in 2010. Regarded as Japan’s flagship intervention in the context of health aid,[14] the handbook has been adopted in other countries, such as Indonesia and Bangladesh,[10, 15] and previous studies have evaluated its impact on perinatal health.[11] However, a high-quality study that assesses the effectiveness of the handbook to facilitate long-term information-sharing has not previously been undertaken.[16]

The World Health Organization (WHO) emphasises the importance of effective interventions that focus on delivering a continuum of care.[8] The MCH handbook facilitates continuum of care throughout pregnancy, delivery and postpartum as well as the child’s infancy using the handbook’s continuous record of basic educational information that encompasses antenatal care and the milestones of child development from the ages of 0–6 years. Women use the handbook by filling out relevant sections with their personal, maternal and child health information, and bringing the handbook with them to all antenatal and postnatal appointments. At the appointment, the midwife and/or doctor then check the relevant section of the handbook pertaining to the woman’s stage of pregnancy or the child’s stage of development, and record in it results of tests, such as protein in the urine during pregnancy, or other notes. The handbook also contains information on MCH care and serves as a valuable communication and educational tool between pregnant women and healthcare professionals, through which women can raise specific health concerns and healthcare professionals can convey important health messages and guidance at point of care.[10]^,^[14] In this study, we aim to measure improved health-seeking behavior by increased antenatal clinic attendance in the Mongolian province of Bulgan. The effectiveness of the intervention will be investigated through a cluster randomized control trial evaluating antenatal attendance, maternal physical and mental health, neonatal health and healthy behaviors. Implementation of the MCH handbook—a communication tool between women and healthcare professionals—can only be conducted at cluster level, and therefore a cluster-randomized trial was employed.

## Methods

### Study design

A cluster-randomized controlled study[17, 18] was conducted from 1 May 2009 until 1 September 2010 among pregnant women and their infants who lived in Bulgan, Mongolia. The allocation ratio was 9/9 = 1.00 and the unit of randomisation in this study was the *soum*—a small administrative unit in Mongolia—and the *bag*, which is a subdivision of a *soum*.

### Participants/ Study population

Eligible participants included pregnant women living in the Bulgan province of Mongolia. Health centres in Bulgan are located in each *soum* and all women must register their newborn infants at their local health centre, regardless of the infant’s birthplace. Data confidentiality was strictly maintained throughout all steps of this study.

### Randomisation and masking

*Soums* and *bags* were selected for administrative convenience and to avoid contamination. Bulgan province is comprised of 17 *soums* and 4 *bags* and they differ in size, health outcomes, and available healthcare facilities. Of the combined *soums* and *bags* (21), 18 units (16 *soums* and 2 *bags*) were selected for inclusion in this study, and randomized in equal number between intervention and control group. Three units were excluded because one *soum* was the subject of a pilot study, and two *bags* were included in another health promotion project. Randomisation was conducted using shuffled, sealed envelopes, and an envelope was selected by each *soum* representative. Since the unit of randomisation is a *soum* and the intervention is visible, the intervention and outcomes could not be masked. Written informed consent was sought from all women for permission to use the collected data in the study.

### Interventions

The Mongolian edition of the handbook was translated into Mongolian from the original Japanese version. The MCH handbook contained a log for recording information on maternal health and personal information, course of pregnancy, delivery and postpartum health, weight during and after pregnancy, dental health, parenting classes, child development milestones from the ages of 0–6 years, immunization and illnesses, and height and weight charts for children. The handbook was used as the intervention at both the cluster and individual participant level. The handbooks were implemented at the beginning of the study observational period, and after a delay of seven months in the control group.

### Outcomes

The primary outcome was the number of antenatal care visits and the proportion of women who made six antenatal care visits or more. In Mongolia, the national standard of antenatal care visits is a minimum of six. Healthcare professionals working in each cluster recorded each antenatal visit for their *soum*. Data was collected for all participants at one month postpartum. Secondary outcomes included clinical outcomes (mortality and morbidities) of women and their infants, as well as healthy behaviors of women and their families.

Characteristics and other outcomes for mothers and their infants were collected 28 days after childbirth via self-reported questionnaires and interviews conducted by trained data collectors. The data collectors visited the family clinic or regional hospital as well as the household to undertake routine check-ups of mothers and infants using a questionnaire. All mortality and morbidity ratios are derived from routinely collected national statistics[19] using the ICD-10 classification system.

### Statistical analyses

The primary analyses followed the intention-to-treat principle and compared the proportion of women who visited health centres for antenatal check-ups and their number of visits between the intervention and control groups. In the analysis of a cluster-randomized trial, correlations of the outcomes of participants in the same cluster should be adequately adjusted.[20] To do this, the generalized estimating equations (GEE) method was adopted to estimate mean difference, risk ratio and risk difference as a measure of the effect, and to calculate their 95% confidence intervals (CI). Multivariate GEE analyses was performed to adjust for possible effects of baseline variables. To quantify household wealth status, principle component analysis was used according to the procedure outlined in the Demographic and Health Survey (DHS) guidelines.[21] The whole population sample in the Bulgan province of Mongolia was used to create the wealth index.

The sample size was determined to detect one mean difference in antenatal care visits between the two groups with a two-sided alpha level of 0.05 and 80% power. Assuming 0.01 intra-cluster correlation, it was estimated that approximately 500 women were required in total.

All statistical analyses were conducted with SAS version 9.2 (SAS Institute, Cary, NC, USA). This clinical trial is registered at the UMIN Clinical Trial Registry (UMIN000001748). Both the protocol and CONSORT checklist of the present trial are presented as S1 and S2 Documents.

### Role of the funding source

This study was supported by the National Center for Global Health and Medicine, Tokyo, Japan. The funding source did not affect the conduct, analyses or results of the study in any way.

## Results

### Baseline characteristics of the soums

**Fig 1:**
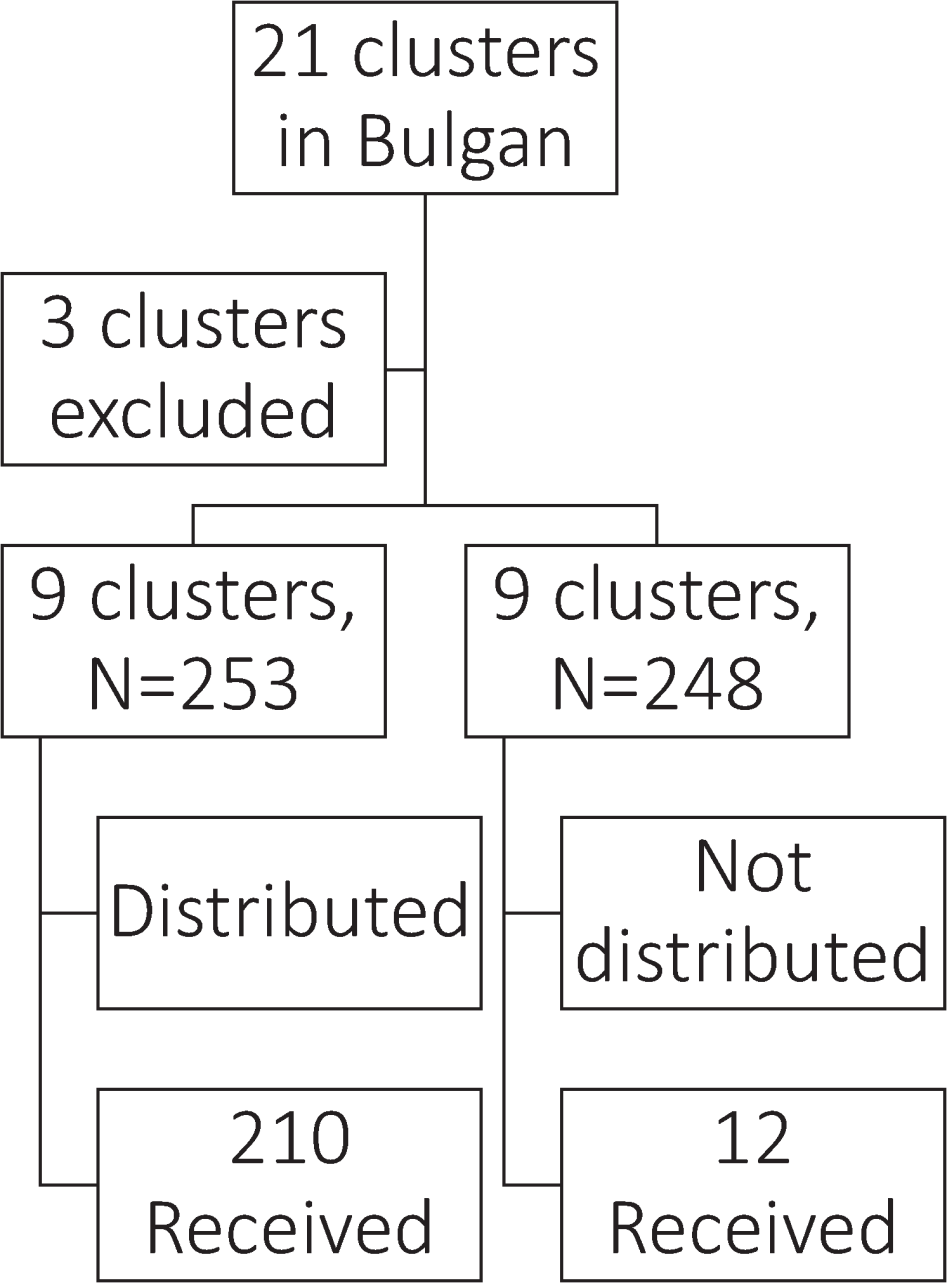
Flow diagram of the study population.

This study had nine clusters in the intervention group and the total intervention population was 253 women with an average of 28·0 people in a cluster. The intervention was implemented between May 2009 and January 2010, and data was collected between February 2010 and August 2010. Of the whole intervention group, only 210 participants received the intervention. There were nine clusters in the control group and the total number of participants for this group was 248 women. There was no difference in the size of the cluster between the two groups. Fig. 1 shows the selection process in detail.

### Baseline characteristics of women and infants

All baseline characteristics were similar in both the intervention and control groups, presented in Table 1.

In this study, 32·4% of participants from the intervention group and 31·0% of the control group were experiencing their first pregnancy; 94·1% of the intervention group and 95·2% of the control group were married and the mean age of both groups was 27 years of age. From the intervention group, 9·5% of participants were educated to elementary school level compared with 10·5% of the control group. Statistically significant differences in travel time were observed between women’s homes and the nearest health centres, (p = 0·008), and in the wealth index (p = 0·001) of the intervention group compared to the control group.

**Table 1:** Baseline characteristics of women and infants.

		Intervention	Control	p-value
		N = 253	N = 248	
First pregnancy	N (%)	82 (32.41)	77 (31.05)	0.743
Number of pregnancies	Mean (SD)	2.49 (1.37)	2.32 (1.24)	0.154
	missing	0	1	
**Outcome of previous pregnancies**				
Live birth	Mean (SD)	1.42 (1.35)	1.29 (1.19)	0.229
Abortion		0.11 (0.41)	0.09 (0.43)	0.556
Miscarriage		0.11 (0.39)	0.07 (0.32)	0.233
Adoption		0.00 (0.00)	0.02 (0.16)	0.099
Pre-pregnancy weight	Mean (SD)	61.10 (9.02)	60.15 (8.76)	0.237
	missing	2	2	
Weight at first antenatal care visit	Mean (SD)	63.13 (9.20)	61.88 (9.19)	0.132
	missing	1	6	
Travel time from home to antenatal care clinic	Median	40	40	0.008
	(25–75%)	(20–99)	(20–60)	
	(min., max.)	(4, 1440)	(2, 180)	
Marital status Married	N (%)	238 (94.1)	236 (95.2)	0.590
Mean maternal age (SD)	Mean (SD)	27.3 (6.13)	27.7 (5.67)	0.390
	missing	1	3	
Maternal educational attainment (up to elementary level education)	N (%)	24 (9.49)	26 (10.48)	0.947
Number of family members in the household	Mean (SD)	4.332 (1.23)	4.185 (1.196)	0.177
Wealth index	Mean (SD)	0.448 (2.194)	-0.225 (2.356)	0.001

### Primary outcome

Women in the intervention group attended antenatal clinics an average of 6·9 times, while women from the control group attended 6·2 times, as shown in Table 2.

**Table 2:** Primary outcome and outcomes for mothers, infants and healthy behaviors.

		Intervention N = 253	Control N = 248	Effect of measure [MD: Mean difference, RR: Risk ratio, RD: Risk difference] (95%CI), p: p-value, *GEE analysis
**Primary outcome**				
Antenatal care visits	Mean (SD)	6.615 (1.525)	6.407 (1.765)	[MD] 0.208 (–0.710–1.125) (p = 0.66)*
Antenatal care visits	≥ 6 N(%)	206 (81.7%)	175 (70.6%)	[RR] 1.158 (0.876–1.532), p = 0.30*, [RD] 11.2% (-9.9%-32.3%), p = 0.30*
**Women’s outcomes**				
Complications identified during pregnancy	N (%)	31 (12.25)	14 (5.65)	P = 0.012
	missing	1	1	
Multiple pregnancies	N (%)	6 (2.37)	4 (1.61)	
Gestational age	Mean (SD)	38.95 (1.25)	39.06 (1.18)	
	Median (25–75%)	39 (38–40)	39 (39–40)	
	missing	7	14	
Cephalic fetal presentation	N (%)	246 (97.23)	236 (95.16)	
Spontaneous vaginal deliveries	N (%)	202 (79.84)	212 (85.48)	
EPDS: Postnatal depression Over cut-off 12 points	N(%)	15 (5.93)	11 (4.44)	RR 0.99 (0.94–1.04), p = 0.560, RD—0.014 (–0.062–0.034), p = 0.561
GHQ: General Health Questionnaire Over cut-off 4	N (%)	3 (1.2%)	5 (2.0%)	RR 1.01 (0.99–1.03), p = 0.412, RD 0.0085 (–0.012–0.029), p = 0.411
**Infant outcomes**				
Apgar score 5 minutes	Mean(SD)	7.55 (0.89)	7.34 (1.25)	MD 0.210 (–0.212–0.632), p = 0.330
	Median (25–75%)	8 (7–8)	7 (7–8)	
	missing	7	6	
Birthweight	Mean(SD)	3388.61(449.00)	3429.11(486.40)	MD–40.50 (–141.53–60.53), p = 0.432
	missing	2	1	
Female	N (%)	123 (48.6)	120 (48.39)	
Any congenital malformation	N (%)	6 (2.37)	3 (1.21)	
Admission of newborn to the Intensive Care Unit	N (%)	6 (2.37)	5 (2.02)	
When did breastfeeding start?	N (%)			RR 1.07 (0.97–1.18), p = 0.186, RD 0.062 (–0.028–0.153), p = 0.176
1) Within one hour after birth		238 (94.07)	217 (87.50)	
2) Between one hour and 24 hours after birth		10 (3.95)	25 (10.08)	
3) After 24 hours		3 (1.19)	2 (0.81)	
4) Breastfeeding not initiated before discharge/ after birth		1 (0.40)	2 (0.81)	
Neonatal status at discharge Death	N (%)	1 (0.40)	2 (0.81)	RR 1.00 (0.99–1.02), p = 0.512, RD 0.0041 (–0.0082–0.016), p = 0.512
**Healthy behaviors**				
Drinking during pregnancy	N (%)	20 (7.91)	35 (14.11)	RR 1.07 (0.97–1.18), p = 0.166, RD 0.061 (–0.024–0.15), p = 0.161
	missing	2	0	
Maternal smoking	N (%)	5 (1.98)	7 (2.82)	RR 1.01 (0.98–1.04), p = 0.572, RD 0.0086 (–0.021–0.038), p = 0.571
	missing	0	1	
Smoking among other members of the household during pregnancy	N (%)	129 (50.98)	151 (60.89)	RR 0.841 (0.71–0.99), p = 0.039, RD −0.097 (−0.194–−0.001), p = 0.048
	missing	1	1	

**Fig 2:**
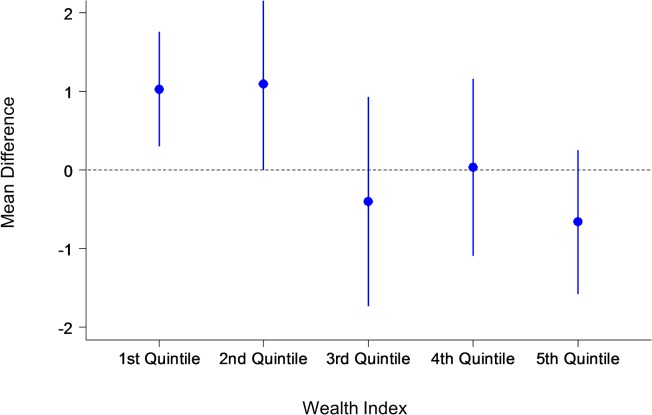
Primary outcome: Mean difference by wealth index. The y-axis shows the mean difference with confidence intervals of the number of antenatal care visits between the intervention and control groups. The x-axis shows the wealth index quintile.

**Fig 3:**
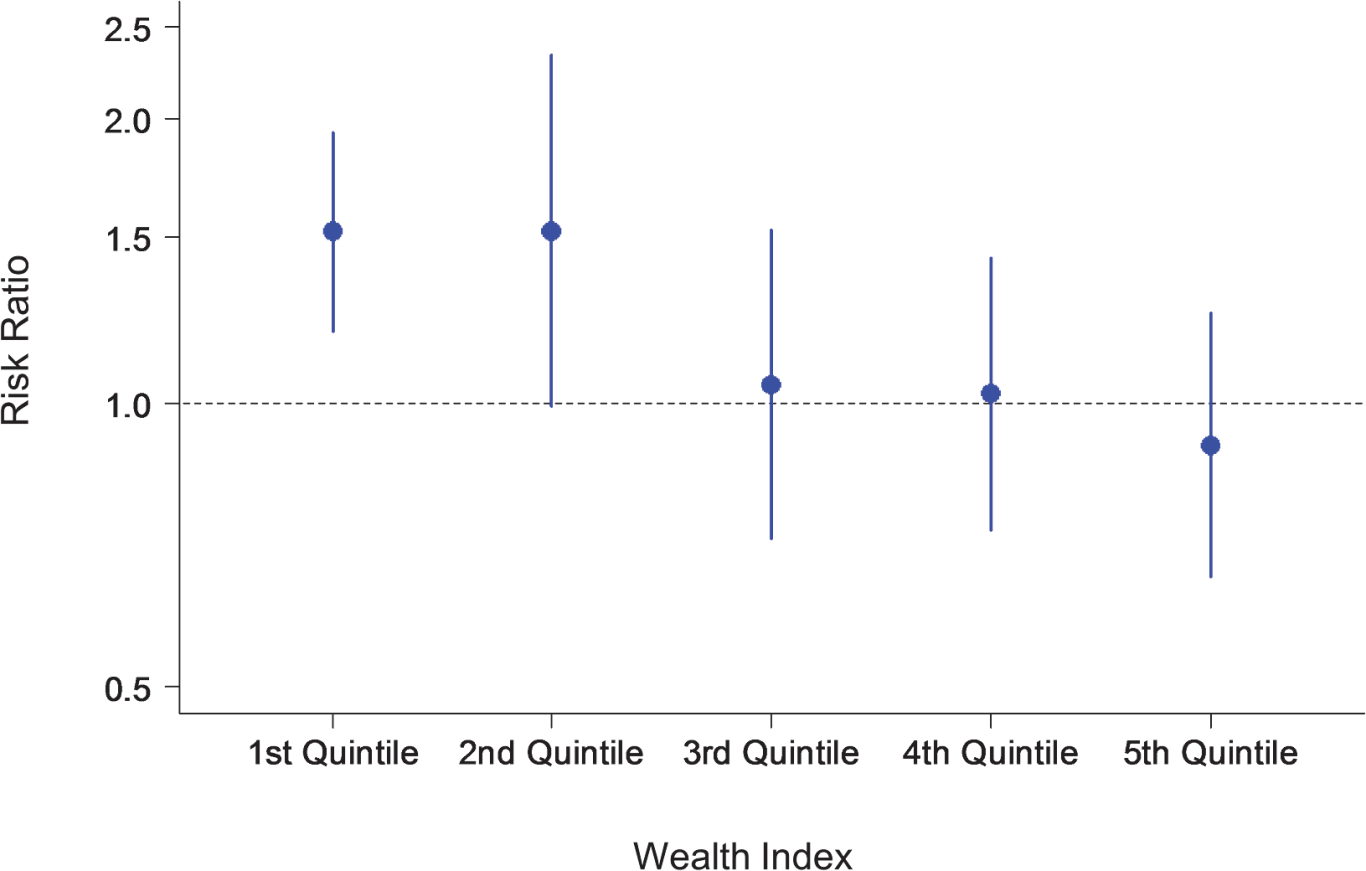
Primary outcome: Risk ratio by wealth index. The y-axis shows the risk ratio with confidence intervals of the number of women who made six antenatal care visits during their pregnancy in the intervention and control groups. The x-axis shows the wealth index quintile.

**Fig 4:**
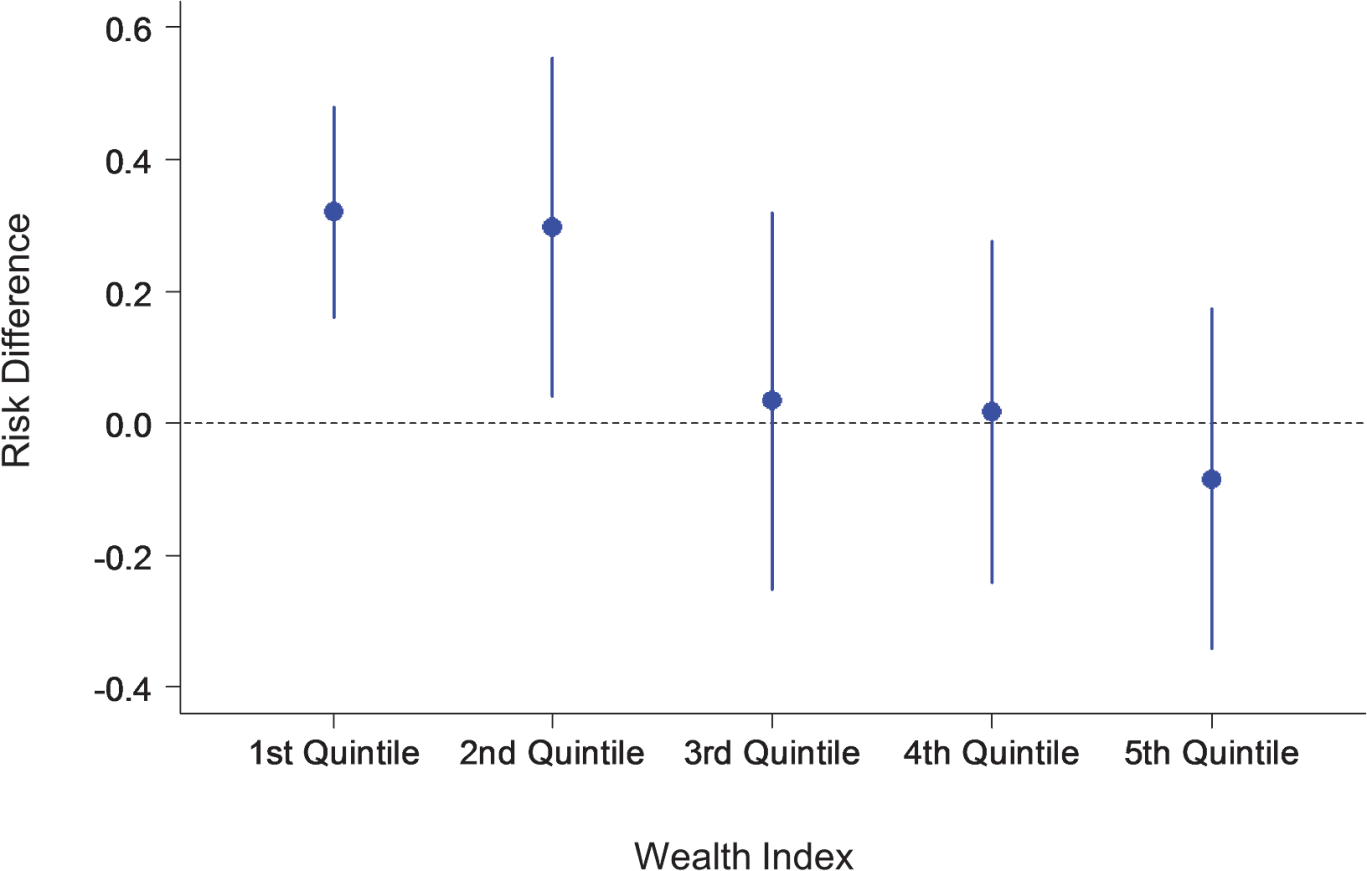
Primary outcome: Risk difference by wealth index. The y-axis shows the risk differences with confidence intervals of the number of women who made six antenatal care visits during their pregnancy in the intervention and control groups. The x-axis shows the wealth index quintile. Note: 1^st^ quintile represents the highest wealth index, and the 5^th^ represents the lowest.

In the primary GEE analysis, there is no significant difference between the two groups in the number of antenatal care visits and the proportion of women who have visited more than 6 times. The travel time to antenatal clinics did not significantly affect the association between the intervention and the primary outcome. However, socioeconomic status was found to influence the frequency of clinic attendance: women of a higher socioeconomic status visited antenatal clinics more often than women from a lower socioeconomic background. Socioeconomic status acted as a statistically significant effect modification on outcomes by the multivariate GEE analyses. Therefore the analysis of primary outcomes was stratified by socioeconomic-status quintile. Results of the GEE analysis of primary outcomes stratified by wealth index are presented in Figs. 2, 3 and 4.

### Women’s health

Complications in maternal health were more likely to be identified, with maternal morbidity during pregnancy at 12·3% in the intervention group compared with 5·7% in the control group. This difference was statistically significant (p-value 0·01). No evidence of difference was observed in women who scored both higher than 12 points in EPDS (RR 0.99 [0.94–1.04], p = 0.56) and higher than 4 points in GHQ (RR 1.01 [0.99–1.03], p = 0.41).

### Infant health

A higher rate of early breastfeeding initiation amongst the intervention group was a significant neonatal health outcome. In the intervention group, 94·1% of infants initiated breastfeeding within one hour of childbirth compared to 87·5% of infants in the control group. This difference, though notable, was not statistically significant.

### Healthy behaviors

An increase in healthy behaviors was observed amongst the intervention group. The majority of women stopped drinking alcohol during pregnancy: only 7·9% of women from the intervention group continued to drink alcohol when pregnant compared with 14·1% in the control group. In the intervention group, a statistically significant reduction in smoking was found among women’s family members living in the same household, with 51·0% of family members continuing to smoke during women’s pregnancies compared with 60·9% living with control group participants.

## Discussion

Our findings show that pregnant women who used the MCH handbook increased their number of antenatal visits from the national requirement of six visits to a mean of 6.9 visits, compared to a mean of 6·2 visits in the control group. Socioeconomic background was also found to play a significant role in clinic attendance for both groups. After adjusting for confounders in the GEE model, the intervention effect was statistically significant, but only among the wealthy. Participants in the wealthiest two quintiles were more likely to attend antenatal clinics more than six times. Complications in maternal health were more likely to be detected among pregnant women who used the handbook. Healthy behaviors were also adopted by partners and other family members of pregnant women in the intervention group: the majority of women did not drink alcohol (7·9% in the intervention group compared with 14·1% in the control group), and approximately half of family members stopped smoking at home, thereby reducing the harm of passive smoking for expectant mothers.

To our knowledge, this study is the first to assess the MCH handbook using a cluster-randomized design in Mongolia. This is also the first cluster-randomized controlled trial to assess a Japanese health aid intervention. The MCH handbook provided pregnant women with a useful educational aid that promotes healthcare-seeking behaviors, fosters continuity of care and enhances communication between pregnant women and their healthcare providers. The intervention raised women’s awareness of maternal and child health concerns and prompted them to seek out healthcare, as illustrated by an increase in antenatal visits. Not only does the handbook instigate the delivery of key health messages from healthcare providers to pregnant women during antenatal visits, but also from pregnant women to their families at home. This study used a randomized cluster design and population-based data collection so its results may be more representative of the effectiveness of the MCH handbook in the community; however, several limitations are present. Masking was not possible among care providers, pregnant women and assessors. Recall bias likely exists in the analysis, because data collection was performed at one month after birth. The unbalanced distribution of socioeconomic status acts as an effect modifier. The per-protocol analysis showed a significant increase in women’s clinic attendance, and therefore it is likely that women of a lower socioeconomic status did not receive the handbook.

A systematic review of a similar intervention highlights the potential benefits of giving women their own health record to use during pregnancy.[22] The review included three randomized trials. Though none of the trials assessed the rate of antenatal care visits as an outcome, the intervention resulted in favourable outcomes such as enhancing a mother’s control over her health, and satisfaction with the care provided.[22] A cross-sectional study conducted by Osaki et al showed the MCH handbook increased utilisation of health services and deliveries with trained personnel.[12] Although the rate of antenatal visits is not reported in Osaki et al’s study, the findings of our study are compatible with this and other studies.[22]

A significant outcome of this study was an increase in the proportion of women who attended antenatal care visits among pregnant women who used the MCH handbook. Travel time did not function as an effect modifier; however, socioeconomic background was particularly relevant, with women from a higher socioeconomic background visiting antenatal clinics more often than those of a lower socioeconomic background. The handbook also facilitated the identification of maternal morbidities during pregnancy and minimized passive smoking in the households of intervention group participants. In response to the study’s main findings, the MCH handbook was implemented as part of the national health policy in Mongolia in 2010 soon after the trial was finished, and the results support the policy. However, policies to reach women of lower socioeconomic status are yet to be developed and more research is required to address this issue.

Our study showed the effectiveness of the MCH handbook to promote long-term information sharing through an increase in antenatal clinic attendance among women who used the handbook. The intervention promotes better communication between women and healthcare specialists and acts as a reference point for women to raise particular concerns and questions about their own health at antenatal clinics, while at the same time giving healthcare workers the opportunity to deliver important health messages. The handbook’s role in enhancing long-term information sharing can make an important contribution to maintaining MDGs 4 and 5.

Further interventions are also necessary to specifically target pregnant women from a lower socioeconomic background in outreach efforts that aim to increase antenatal clinic attendance. Future research should also focus on the effectiveness of the handbook in other provinces within Mongolia as well as other low- to middle-income countries, where the handbook can be used as an effective tool in maternal health education to further promote maternal health awareness and healthy behaviors, enable early interventions, reduce adverse birth outcomes in developing settings and sustain the achievement of MDGs 4 and 5. Use of the latest information technology, such as a smartphone application of the MCH handbook to facilitate use of the intervention, may also provide a valuable opportunity to enhance accessibility of the handbook, and would benefit from further research.

## Supporting Information

S1 Document Protocol: Cluster randomised controlled trial of Maternal and Child Health Handbook in Mongolia. (PDF)

S2 Document CONSORT checklist: CONSORT 2010 checklist of information to include when reporting a randomised trial. (DOC)

S3 Document Dataset of the present trial. (XLSX)

S4 Document Data dictionary of the trial dataset. (XLSX)

S5 Document Questionnaire used in the trial. (DOC)
