# Supplementary material for: The Maternal and Child Health (MCH) Handbook in Mongolia: A Cluster-Randomized, Controlled Trial
Source: PLoS One. 2015 Apr 8;10(4):e0119772. doi: 10.1371/journal.pone.0119772 (PMC4390384; doi:10.1371/journal.pone.0119772)
Supplement: S1 Document — (PDF) [file pone.0119772.s001.pdf]

A CLUSTER RANDOMISED CONTROLLED TRIAL OF MATERNAL AND CHILD HEALTH HANDBOOK IN MONGOLIA

Version 2.0 (2010/03/18)

BACKGROUND

Maternal mortality and perinatal mortality ratios in Mongolia are 89 per 100000 and 21 per 1000 childbirths, respectively. Mortalities of older children have been declined, though mother and newborn health remains a major health challenge in Mongolia. Care for women and children starting before pregnancy through childbirth into the babies' childhood has been promoted to achieve health-related Millennium Developmental Goals, especially 4 and 5, as Continuum of Care.[1]

Maternal and child health handbook was firstly developed and implemented in 1947 in Japan, where maternal and neonatal mortality rates have been declined from 160.1 per 100000 births and 31.4 per 1000 births in 1947 to 5.7 and 1.4 in 2005, respectively.[2] Japan has now claimed their successful promotion of maternal and child health with the extremely low perinatal mortality. One of the major features of the maternal and child health handbook is not only to provide the information about growth and development of the child to share it between parents and healthcare professionals, but also to provide continuous record and basic educational information for the family from antenatal care up to milestones of growth and development of these children, which facilitate the Continuum of Care. The handbook has been implemented in other countries, too, such as Indonesia and Bangladesh.[3] The handbook has been evaluated in a previous study.[4] However, there has not been a high-quality study assessing effectiveness of use of such a handbook to facilitate sharing continuous information.

Providing Maternal and Child Health Handbook has cost implication for all children born, and it is extremely important to appropriately assess its effectiveness when firstly initiated or before the implementation.

It also has implication to a better data collecting system to provide evidence-base for policy development in maternal and child health.[5]

AIM AND OBJECTIVES

AIM:

To promote health of women and children especially in high mortality settings

OBJECTIVES:

1) To evaluate effectiveness of Maternal and Child Health Handbook in Mongolia
2) To assess acceptability of Maternal and Child Health (MCH) Handbook by both families and healthcare professionals in Mongolia
3) To investigate socioeconomics and health related status of Mother and Child in Bulgan, Mongolia

STUDY DESIGN:

STUDY TYPE

A cluster randomised controlled trial

TARGER POPULATION

All pregnancy women and their child living in Bulgan aimag

INTERVENTION AND COMPARATOR

The intervention is implementation of MCH Handbooks at the beginning of the study observational period, and implementation of MCH handbook after 1 year of observation serves as a control.

OUTCOME

1) Primary outcomes

Improvement in the number of antenatal care visits

2) Secondary outcomes

Improvement in perception and satisfaction of mother towards maternal and child health care
Improvement in perception and knowledge for MCH handbook and related
Improvement in the number of regular health check-up of infants

Improvement in maternal morbidity rate
Improvement in maternal mortality rate
Improvement in infant morbidity rate
Improvement in perinatal mortality rate
Improvement in infant mortality rate

Edinburgh Postnatal Depression Scale (EPDS)
General Health Questionnaire (GHQ) with 12items

DETAILS AND IMPLEMENTATION OF INTERVENTION

The maternal and child health handbook for Mongolia will be developed adopting from the Japanese by translating it with the new WHO growth chart.

JUSTIFICATION

A randomised controlled trial is so far the best study to prove effectiveness of a public health intervention. As the unit of the intervention, implementation of maternal and child health hand book, is by sums, the best possible study design is a cluster randomised controlled trial. There are 17 soums in Bulgan, Mongolia, though these are different in their size, health outcome index, and care they receive. It is therefore considered that cluster controlled trial with 15 soums and not controlled survey a bug (No 3 bug in Bulgan city) and a soum (NAME) is the best possible option for the research question.

DATA COLLECTION

MORTALITY AND MORBILITY:

All mortality and morbidity are derived from the routinely collected national statistics system by ICD-10 code.

CHARACTERRISTICS AND OTHER OUTCOMES OF MOTHERS AND CHILD:

This is collected by using questionnaire by interview after 28 days at birth. The data collectors for survey visit the family clinic or Aimag hospitals and the household with mother in routine check-up for children.

STUDY IMPLICATION

There are three major implications expected from this study:

1) Distribution of maternal and child health handbook for all pregnancies has cost implication, and the results will inform the Mongolian government to make informed judgement whether this is to be implemented for all sums continuously after the study is finished.
2) Strengthen evidence-base development to make informed judgement in health policy in Mongolia
3) The results can be applied to any other countries with similar social and economic status

ETHICAL CONSIDERATION

The intervention itself has been implemented in many other countries, though it has not been done in Mongolia. There is an issue of delaying implementation of MCH Handbook in the controlled group, though implementing such public health interventions will take a time, and delay of one year will be justified.

Confidentiality of the data should be secured in any process of this study, and ethical approval should be obtained by either or both the Mongolian Government and Osaka Medical Center and Research Institute for Maternal and Child Health.

RESEARCH TEAM

PRINCIPLE INVESTIGATORS

Samdan Dulamsuren

Deputy Director, National Center for Health Development, MOH

Oyun Lkhagvasuren

National Center for Health Development, Ministry of Health, Mongolia

Rintaro Mori

Osaka Medical Center and Research Institute for Maternal and Child Health, Japan

Yasuhide Nakamura

Osaka University, Japan

Gochoo Soyolgerel

Ministry of Health, Mongolia

Naohiro Yonemoto

Osaka Medical Center and Research Institute for Maternal and Child Health, Japan

TIMELINE

The overall framework of the study is shown below:

Jan 2009	Kick off meeting (randomization) and MCH handbook workshop
May 2009	Implementation of MCH handbook start
Jan 2010	Data collection workshop and MCH handbook workshop
March 2010	Data collection workshop and survey start
July 2010	Data check work shop
Sep 2010	Data collection workshop and survey close

LOGISTICS

18 soums will be selected for this study. All data collectors will selected from local soums medical stuff (each 2 persons). The data checker will selected from Aimag health ceter (2 persons) The data management and analysis will take place in Bulgan aimag, Ulaanbaatar city, Mongolia and Japan. The report will be published in Mongolian and

English. The dissemination workshop will include representatives from local communities involved in the study, representatives from governmental, non-governmental and international organizations.

The local logistics will be taken care by the staff of the National Center fro Health Development.

RESEARCH GRANT

All the research grant is from International Cooperation Research Grant by Ministry of Health, Labour and Welfare

REEFRENCES

1. Kerber, K.J., et al., *Continuum of care for maternal, newborn, and child health: from slogan to service delivery.* Lancet, 2007. **370**(9595): p. 1358-69.
2. Mamiya, U., *Japan's Maternal and Child Health Handbook.* Midwives Chron, 1990. **103**(1233): p. 314-5.
3. Fujimoto, S., et al., *[Utilization of Maternal and Child Health handbook in Japan].* Nippon Koshu Eisei Zasshi, 2001. **48**(6): p. 486-94.
4. Takayanagi, K., S. Iwasaki, and Y. Yoshinaka, *The role of the Maternal and Child Health Handbook system in reducing perinatal mortality in Japan.* Clin Perform Qual Health Care, 1993. **1**(1): p. 29-33.
5. Kunugi, H., et al., *Perinatal complications and schizophrenia. Data from the Maternal and Child Health Handbook in Japan.* J Nerv Ment Dis, 1996. **184**(9): p. 542-6.
