# Supplementary material for: The Maternal and Child Health (MCH) Handbook in Mongolia: A Cluster-Randomized, Controlled Trial
Source: PLoS One. 2015 Apr 8;10(4):e0119772. doi: 10.1371/journal.pone.0119772 (PMC4390384; doi:10.1371/journal.pone.0119772)
Supplement: S5 Document — (DOC) [file pone.0119772.s005.doc]

Mongolia Maternal Child Health Handbook Study

Individual form

(For Mother and Child)

Version 1.5 (2010/03/05)

**Identification**

Name of soum living in the mother _________________

Name of delivery place (Health Facility) _________________

Mother national registration number _________________

Child national registration number _________________

**(This page data collects from hospital records)**

(If the participant has **twin and more,** the second go to the second page in **Delivery Data,** section)

**Pregnancy of the woman**

1. The latest pregnancy was your first pregnancy 1) Yes　 2) No

2. Number of pregnancies, including the latest one pregnancy __ __

3. Outcomes of previous pregnancies

1) live birth, how many? __ __ 2) abortion, how many? __ __

3) miscarriage, how many? __ __ 4) adoption, how many? __ __

4. Total number of antenatal visits during latest pregnancy 　 __ __

1st Visit (YY/MM/DD) __ __ /__ __/ __ __ 2nd Visit (YY/MM/DD) __ __ /__ __/ __ __

3rd Visit (YY/MM/DD) __ __ /__ __/ __ __ 4th Visit (YY/MM/DD) __ __ /__ __/ __ __

5th Visit (YY/MM/DD) __ __ /__ __/ __ __ 6th Visit (YY/MM/DD) __ __ /__ __/ __ __

7th Visit (YY/MM/DD) __ __ /__ __/ __ __ 8th Visit (YY/MM/DD) __ __ /__ __/ __ __

9th Visit (YY/MM/DD) __ __ /__ __/ __ __ 10th Visit(YY/MM/DD) __ __ /__ __/ __ __

5. Date of last menstruation

As mother said (YY/MM/DD) __ __ /__ __/ __ __

By ultrasound (YY/MM/DD) __ __ /__ __/ __ __

1. Pre-pregnancy weight __ __ __kg, Height __ __ __ cm
2. First antenatal care visit weight __ __ __kg
3. Travel time from woman’s home to antenatal care clinic __ __ minutes
4. Syphilis testing

First syphilis testing Reactive & Confirmed / Not reactive / Unknown/No test

Testing day (YY/MM/DD) __ __ /__ __/ __ __

　Second syphilis testing Reactive & Confirmed / Not reactive / Unknown/No test

Testing day (YY/MM/DD) __ __ /__ __/ __ __

　Third syphilis testing Reactive & Confirmed / Not reactive / Unknown/No test

Testing day (YY/MM/DD) __ __ /__ __/ __ __

Travel time from woman’s home to syphilis screening clinic __ __ minutes

Newborn diagnosed with congenital syphilis 1)Yes 　2)No

1. History of disease before pregnancy 1)Yes 2) No

　　If Yes, 1) Sexually transmitted infection, specify ________________

2) Other ________________________

1. Admission of the mother to ICU/Special care unit during the latest pregnancy 1) Yes, 2) No, If yes, how many times? __ __
2. Total number of days spent in Intensive/Special care unit __ __ days
3. Date of maternal discharge from the hospital

(YY/MM/DD) __ __ /__ __/ __ __

**(This page data collects from hospital records)**

**Delivery Data**

1. Date of delivery (not birth date) (YY/MM/DD) __ __ /__ __/ __ __
2. Multiple births 1) No, single 2) Yes, twin 3) Yes, triple or more __
3. Best obstetric estimate of gestational age at delivery (in completed weeks)

__ __ weeks

1. Fetal presentation at delivery 　1) Cephalic 2) Breech 3)Other ____________
2. Final mode of / assistance for delivery

1) Spontaneous 2) Forceps extraction

3) Vacuum extraction 4) Elective C-section no labor

5) Emergency C-section no labor 6) Intrapartum C- Section

7) Assisted breech or breech extraction 8) Internal version and extraction

9) Laparotomy for uterus rupture

1. Mother’s weight of delivery day __ __ __ kg

**Infant data**

1. Status of birth 1) Alive 2) Fresh stillbirth 3) Macerated stillbirth
2. Apgar score at 5 minutes ______
3. Birth weight __ __ __ __ g
4. Body height __ __ cm
5. Infant sex 1) Female, 2)Male
6. Any congenital malformation 1) Yes, 2) No

If yes, name of disease and code ________________________

1. Admission of the newborn to ICU/ Special care unit

1) No, 2) Yes, not ventilated 3) Yes, ventilated

If, 2), 3) Yes,

Total number of days spent at the Intensive/ Special Care Unit __ __ days

1. Newborn status at discharge from the hospital

1) Alive and well

2) Alive with obstetric trauma

3) Alive but referred to a high level or special care unit

4) Dead within 24 hours of birth

5) Dead after 24 hours of birth

1. When was breast feeding started?

1) Within one hours after birth

2) Between one hours and 24 hours after birth

3) After 24 hours

4) Breast feeding not initiated before discharge after birth

1. Neonatal Status and date of neonatal discharge from the hospital or transfer or

death

1. Alive 2) Dead
2. Discharge or Dead date (YY/MM/DD) __ __ /__ __/ __ __

**(This page data collects from interview from mother)**

(If the participant has twin and more, the second go to the Last page)

**Personal data for a mother**

1. Marital status

1) Single 2) Married/cohabitating

3) Separated/divorce 4) Windowed/other

1. Mother’s age ____ ____
2. Education of mother

　1) Uneducated 　　2) Elementary　　　3) Incomplete secondary

4) Complete secondary　　5) Incomplete high

6) High (completed collage or university)

1. Number of family member ____ ____
2. Living condition 1) Ger 2) House 3) Apartment

4) Other, specify ____________

34-1.　Number of rooms or ger wall ____ ____

34-2. Ownership of dwelling 1) Own 2) Other, specify_____________

34-3. Does your household have?

Electricity 1)Yes　　2)No

Television 1)Yes　　2)No

Computer 1)Yes　　2)No

Mobile telephone 1)Yes　　2)No

Refrigerator 1)Yes　　2)No

34-4. Does any member of your household own:

Motorcycle/Scooter 1)Yes　　2)No

Animal drawn-cart 1)Yes　　2)No

Tractor 1)Yes　　2)No

Car 1)Yes　　2)No

34-5. Does any member of this household own any land that can be used for agriculture?

1) Yes, ____m^2^ 2) No

34-6. Does this household own any livestock, herds, or farm animals?

1) Yes 2) No

how many Cows___ ___ Horses___ ___ Goats ___ ____ Sheeps___ ___ Camels___ __ Others (specify)____ ____

34-7. What is main source of drinking water for your household?

1) Tap/piped water in residence 2) Outside tap (piped water)

3) Public tap 4) Well-water, within residence (private well)

5) Outside/public well 6) Spring water

7) River/stream/pond/lake/dam 8) Rainwater

9) Tanker/truck/water vendor 10) Other (specify)____

34-8 What kind of toilet facility does your household have?

1) Own flush toilet 2) Shared flush toilet

3) Ventilated improved pit latrine 4) River/canal

5) No facility/bush/field 6) Other(specify)____

34-9 What are the main materials used in the roof?

1) Roof from natural materials 2) Rudimentary roof

3) Tiled or concrete roof 4) Corrugated iron

34-10 Covering of ger 1) Single 2) Double

1. How many people live in your home? ____ ____
2. How many do you have support person for bearing your child? ___ ___
3. Local resident 1) Yes 2) No, specify ____________
4. Religion of the head of this household? 1) Buddhism

2) Other religion (specify) ____________

3) No religion

**(This page data collects from interview from mother)**

**Mother’s drinking, smoking and eating**

1. Did you drink alcohol during pregnancy? 1)Yes　　2)No

Drink Definitions a drink is defined as follows

A single small (8 ounces; 1/2 pint) glass of beer,

a single shot/measure of liquor/spirits, and a single glass of wine.

If YES answer following questions.

39-1 How often do you have a drink containing alcohol?

1) Never 2)Monthly or less 3) Two to four times per month

4) Two to three times per week 5) Four or more times per week

39-2 How many drinks containing alcohol do you have on a typical day when you

are drinking?

1) 1 or 2 2) 3 or 4 3) 5 or 6 4) 7, 8 or 9 5)10 or more

39-3 How often do you have 6 or more drinks on one occasion?

1) Never 2) Less than monthly 3) Monthly 4) Weekly 5) Daily or almost daily

1. Have you ever been a smoker? 1)Yes　2)No

If Yes,

- 1. How many cigarettes per day? ( )/day
  2. When did you smoke?

1) Quite before conception 2) First trimester 3) Second trimester,

4) Third trimester 5) postpartum

1. During your pregnancy, was there any period of time when you lived with someone who smokes?

1) Yes 2) No 3) Do not know

1. How often do you eat fish?

1) Never 2) Monthly or less 3) Monthly 4) Weekly or 2 days per week

4) 3 or 4 days per week 5) 5 days or more per week

**(This page data collects from interview from mother)**

**Knowledge and Satisfaction**

*Now I am going to ask some questions about the antenatal visits that you have had during this pregnancy*

1. Have you had antenatal checkups during your last pregnancy?

1) Yes 2) No

1. Are you happy about the number of antenatal checkups you have had, or would you have preferred

1) more check-ups 2) fewer check-ups 3) number of check-ups was right

1. Has the time between checkups been:

1) too short 　　　　2) too long 　　　　3) about right

1. How long do you usually have to wait at the unit (clinic /hospital) before being seen by a doctor/ nurse / midwife who provides you antenatal care? __ __ minutes
2. Are you happy with the time you normally have to wait? 1) Yes 2) No
3. How much time do you usually spend with the doctor/ nurse /midwife who provide you antenatal care ? __ __ minutes
4. Do you have enough time with the doctor/ nurse during you checkups, or would you prefer

1) a lot more time 2) a little more time 3) time is about right

1. If you had a choice, would you prefer to be seen by:

1) a male provider 2) a female provider 3) no preference

- 1. If you had a choice, would you prefer to be attended by:

1) a doctor 2) a nurse 3) a midwife 4) no preference

**(This page data collects from interview from mother)**

*Now I am going to ask you more about the care you have had. First some questions about the information you received from the doctors and nurses who provided you with antenatal care.*

51. Have you received any information from your doctor or nurse about your health?

1) Yes 2) No

52. Was the information you received about looking after you own health

1) not enough 2) as much as you wanted 3) too much

4) no information received 5) don’t remember

53. Was the information you received about the tests (e.g. blood, urine) during this

pregnancy:

1) not enough 2) as much as you wanted 3) too much

4) no information received 5) don’t remember

54. Was the information you received about treatment you might need during this

pregnancy

1) not enough 2) as much as you wanted 3) too much

4) no information received 5) don’t remember

55. Was the information you received about labor

1) not enough 2) as much as you wanted 3) too much

4) no information received 5) don’t remember

56. Was the information you received about breastfeeding

1) not enough 2) as much as you wanted 3) too much

4) no information received 5) don’t remember

57. Was the information you received about family planning

1) not enough 2) as much as you wanted 3) too much

4) no information received 5) don’t remember

58. Was the information you received about vaccination about infant

1) not enough 2) as much as you wanted 3) too much

4) no information received 5) don’t remember

**(This page data collects from interview from mother)**

**Antenatal care and MCH handbook**

59. In general, how satisfied are you with the antenatal care you have received so far in this unit (clinic/ hospital)

1) Very satisfied 2) Satisfied 3) Not satisfied

60. Did you think antennal care is needed?

1) Yes 2) No

61. Have you received the maternal and child health handbook?

1) Yes, I have 2) Yes, but I lost it 3) Yes, but I lost and received it again

4) No

If you select 1) 2) 3) Yes,

When did you received it 　 (YY/MM/DD) 　__ __ /__ __/ __ __

Where did you received it (name of place, hospital) 　________________

Who did provide to you 　 ________________

62. In general, how satisfied are you with the maternal and child health handbook you have received

1) used, very satisfied 2) used, satisfied 3) used, not satisfied,

4) received, not used 5) not received

63. Do you think the maternal and child health handbook has necessary information that you needed?

1) Yes 2) No

64. Do you know the word of “Growth Chart”

1) Yes 2) No

If Yes,

64-1. Have you ever seen it before ?

1) Yes 2) No 3) I don’t know

64-2. Have you ever used it before ?

1) Yes 2) No 3) I don’t know

**(This page data collects from interview from mother)**

**Knowledge about the infection and antenatal syphilis screening**

65. Do you know a disease “syphilis”? 1) Yes 2) No

66. Is syphilis harmful for a pregnant woman’s health?

1) Yes 2) No 3) I do not know

67. Is syphilis dangerous for an unborn baby? 1) Yes 2) No 3) I do not know

68. Do you give a test for syphilis infection during pregnancy?

1) Yes 2) No 3) I do not know

69. Is the blood test for syphilis necessary among pregnant women?

1) Yes 2) No 3) I do not know

**Mother’s knowledge and attitude for vaccination**

1. Did your baby receive any vaccine after birth? 1) Yes 2) No 3) I do not know

If yes, why did you receive vaccine to your baby?

1. With high benefits 1) Yes 2) No
2. Pediatrician recommends it 1) Yes 2) No
3. No problem in the past 1) Yes 2) No
4. Avoid feeling regret if baby become ill because not vaccinated 1) Yes 2) No
5. Everyone does it 1) Yes 2) No
6. Do you know age of your baby at initiation of routine schedule?

1) At birth day (within 24 hours after birth) 2) Second day of the bith

3) 3-7 days 4) 1 moth 5) 2-3 months

6) 4-6 months 7) Older than 6 months

1. Are vaccines necessary?

1)Yes 2) No 3) I do not know

1. Do you want to receive information about on vaccines?

1) Yes 2) No, I do not need 3) No, I trust my doctor

1. Do you worry about vaccination?

1) Yes 2) No

If yes,

1. Worry vaccine will not work 1) Yes 2) No
2. Afraid doctor will give wrong vaccine 1) Yes 2) No
3. Afraid “they” are experimenting when they give vaccines 1) Yes 2) No
4. Diseases that vaccines prevent are not bad 1) Yes 2) No
5. If everyone else’s baby is vaccinated then mine does not need to be 1) Yes 2) No
6. Trust regarding vaccination. Please choose one closest answer.
   1. Afraid doctor will give wrong vaccine
7. Strongly disagree 2) disagree 3) Somewhat disagree

4) Not at all 5) Somewhat agree 6) Agree 7) Strongly agree

- 1. Afraid “they” are experimenting when they give vaccines

1. Strongly disagree 2) disagree 3) Somewhat disagree
2. Not at all 5) Somewhat agree 6) Agree 7) Strongly agree
   1. Do not trust information that the doctor give about vaccines
3. Strongly disagree 2) disagree 3) Somewhat disagree
4. Not at all 5) Somewhat agree 6) Agree 7) Strongly agree
   1. Pediatrician is only allowed to tell me information about vaccines in a way that makes risks of vaccines seem low
5. Strongly disagree 2) disagree 3) Somewhat disagree
6. Not at all 5) Somewhat agree 6) Agree 7) Strongly agree
   1. Pediatrician does not have time to talk with me about vaccines
7. Strongly disagree 2) disagree 3) Somewhat disagree
8. Not at all 5) Somewhat agree 6) Agree 7) Strongly agree
   1. Pediatrician does not want me to ask a lot of questions about vaccines
9. Strongly disagree 2) disagree 3) Somewhat disagree
10. Not at all 5) Somewhat agree 6) Agree 7) Strongly agree
    1. Comfortable talking to pediatrician about vaccines
11. Strongly disagree 2) disagree 3) Somewhat disagree
12. Not at all 5) Somewhat agree 6) Agree 7) Strongly agree
    1. Reason to vaccinate is that the pediatrician recommends it
13. Strongly disagree 2) disagree 3) Somewhat disagree
14. Not at all 5) Somewhat agree 6) Agree 7) Strongly agree
    1. Doctors are supportive of my worries about vaccination
15. Strongly disagree 2) disagree 3) Somewhat disagree
16. Not at all 5) Somewhat agree 6) Agree 7) Strongly agree

**(This page data collects from interview from mother)**

**Infant feeding**

1. At the moment is your baby…

1) Breast fed →Go to Q75-1 2) Bottle fed → Go to Q76

3) Both → Go to Q78

76-1 Do you ever give your baby milk in a bottle at present

(apart from expressed breast milk)?

1) Yes (even if only occasionally) → Go to Q77

2) No → Go to Q79

1. Did you ever put your baby to the breast?

1) Yes (even if it was once only) → Go to Q77

2) No never → Go to Q78

1. How old was your baby when last breast fed him/her ____________days/week
2. Which kind of milk do you give your baby most of the time at the moment?

1) Formula 2) Liquid cow’s milk → go to Q78-1

3) Another kind of milk (please write in the name) →go to Q78-1

78-1. If you use cow’s milk, is it

1) Whole 2) Semi-skimmed 3) Skimmed

78-2. Do you ever add anything to the baby milk in the bottle?

1) Yes → go to 77-3 2) No → go to Q78

78-3. What do you add to the baby milk?

1) Sugar 2) Honey 3) Tea 4) Other _____________

1. In which position do you usually place your baby to sleep?

1) On his/her back 2)On his/her front 3)On his/her side 4)Varies

1. How would you feel your baby’s health. Is it

1) Excellent 2)Very good 3) Good 4)Fair 5)Poor 6)Do not know

**(This page data collects from interview from mother)**

( Edinburgh Postnatal Depression Scale (EPDS)　）

Instructions for users:

1 The mother is asked to underline the response which comes closest to how she has been feeling in the previous 7 days.

2 All ten items must be completed.

3 Care should be taken to avoid the possibility of the mother discussing her answers with others.

4 The mother should complete the scale herself, unless she has limited English or has difficulty with reading.

********************************************************************************

As you have recently had a baby, we would like to know how you are feeling. Please write CIRCLE the answer which comes closest to how you have felt IN THE PAST 7 DAYS, not just how you feel today.

1. I have been able to laugh and see the funny side of things.

1) As much as I always could

2) Not quite so much now

3) Definitely not so much now

4) Not at all

2. I have looked forward with enjoyment to things.

1) As much as I ever did

2) Rather less than I used to

3) Definitely less than I used to

4) Hardly at all

3. I have blamed myself unnecessarily when things went wrong.*

1) Yes, most of the time

2) Yes, some of the time

3) Not very often

4) No, never

4. I have been anxious or worried for no good reason.

1) No, not at all

2) Hardly ever

3) Yes, sometimes

4) Yes, very often

5. I have felt scared or panicky for not very good reason.*

1) Yes, quite a lot

2) Yes, sometimes

3) No, not much

4) No, not at all

6. Things have been getting on top of me.*

1) Yes, most of the time I haven't been able to cope at all

2) Yes, sometimes I haven't been coping as well as usual

3) No, most of the time I have coped quite well

4) No, I have been coping as well as ever

7. I have been so unhappy that I have had difficulty sleeping. *

1) Yes, most of the time

2) Yes, sometimes

3) Not very often

4) No, not at all

8. I have felt sad or miserable.*

1) Yes, most of the time

2) Yes, quite often

3) Not very often

4) No, not at all

9. I have been so unhappy that I have been crying.*

1) Yes, most of the time

2) Yes, quite often

3) Only occasionally

4) No, never

10. The thought of harming myself has occurred to me.*

1) Yes, quite often

2) Sometimes

3) Hardly ever

4) Never

**(This page data collects from interview from mother)**

(General Health Questionnaire)

Please consider the last four weeks and answer the following questions by selecting and circling one of the four answer options.

| Question | 1 | 2 | 3 | 4 |
| --- | --- | --- | --- | --- |
| 1. Been able to concentrate on what you’re doing | Better than usual | Same as usual | Less than usual | Much less than usual |
| 2. Lost much sleep over worry | Not at all | No more than usual | Rather more than usual | Much more than usual |
| 3. Felt you were playing a useful part in things | More so than usual | Same as usual | Less useful than usual | Much less useful |
| 4. Felt capable of making decisions about things | More so than usual | Same as usual | Less useful than usual | Much less useful |
| 5. Felt constantly under strain | Not at all | No more than usual | Rather more than usual | Much more than usual |
| 6. Felt you couldn’t overcome your difficulties | Not at all | No more than usual | Rather more than usual | Much more than usual |
| 7. Been able to enjoy your normal day-to-day activities | More so than usual | Same as usual | Less useful than usual | Much less useful |
| 8. Been able to face up to your problems | More so than usual | Same as usual | Less useful than usual | Much less useful |
| 9. Been feeling unhappy and depressed | Not at all | No more than usual | Rather more than usual | Much more than usual |
| 10. Been losing confidence in yourself | Not at all | No more than usual | Rather more than usual | Much more than usual |
| 11. Been thinking of yourself as a worthless person. | Not at all | No more than usual | Rather more than usual | Much more than usual |
| 12. Been feeling reasonably happy, all things considered | More so than usual | About the same as usual | Less so than usual | Much less than usual |

COMMENTS

-------------------------------------------------------------------------------------------------------------------------

Interviewer’s name ____________

Date of interview (MM/DD/YY) __ __ /__ __/ __ __

Signature ____________
